# Selecting the Shade

**Published:** 1904-07

**Authors:** Geo. W. Schwartz

**Affiliations:** Chicago.


### SELECTING THE SHADE.

By Geo. W. Schwartz, M. D., D. D. S., Chicago.

(Written for *The American Journal of Dental Science.*)

While the subject of selecting the shade in inlay work is not an extensive one it is an important one.

In the use of the shade guide all matching should be done before the rubber dam is adjusted. After selecting the shade in a natural light, compare it also in an artificial light, with the room previously darkened, if you wish to be critical. Then view the shade from both sides of the chair in various angles.

Experience teaches that porcelain fillings for the mesial surfaces of the eight anterior teeth should be a shade darker than the tooth, while those for the distal should be a shade lighter.

For second bicuspids and molars it is safe to bake from one to two shades darker as a rule, with few exceptions. In centrals and laterals it is a safe rule to use white as a foundation, finishing with the colors desired, while nearly all posterior to these should have a yellow foundation. The color of the cusps in almost all bicuspids and molars incline to blue, with few exceptions. In most teeth the cusps are lighter at the point and the body should be built on, with this fact in mind.

The color of the cement should not be over-looked by beginners. It is a suggestion on my part to begin by the use of one color cement and that a yellow, at the most not more than two, a yellow and a blue. It is not advisable for the beginner to depend on his ability to select the color of his cement to help the shade of his inlay; if he does he will meet with many disappointments, for instance: an inlay we will say for a yellow tooth that matches it almost perfectly before it is cemented, after being cemented in place with a yellow cement that matches, will make the inlay or filling appear from one to two shades darker than before it was cemented. The same is true of the blue shade. For this reason it is my belief the beginner can better learn to shade his porcelain to the cement than to depend on the selection of the cement for the matched inlay. I wish to say this statement is not entirely original with me, as it was advanced by Dr. Cheeseman also.

In working colors for cusp restoration in molars and bicuspids, I find the use of dark brown and dark yellow in the fissures, as the case may require, results in good effects. I have had very satisfactory and artistic results in the use of oil colors for the fissures and buccal margins and unhesitatingly recommend them to beginners.

As a safe plan I would advise baking a button from each bottle of inlay material and gluing it to a white card, then pasting it to the bottle. While shades run reasonably true, it is very convenient to know the exact shade of each bottle.

I understand this article is for beginners, so I will not confuse them with further details.

---

### FUSING THE PORCELAIN.

By J. E. Hancock, D. D. S., Chicago.

(Written for *The American Journal of Dental Science.*)

